# LSD1 inhibition modulates transcription factor networks in myeloid malignancies

**DOI:** 10.3389/fonc.2023.1149754

**Published:** 2023-03-10

**Authors:** Emily E. Hartung, Kanwaldeep Singh, Tobias Berg

**Affiliations:** ^1^ Centre for Discovery in Cancer Research, Faculty of Health Sciences, McMaster University, Hamilton, ON, Canada; ^2^ Department of Biochemistry and Biomedical Sciences, Faculty of Health Sciences, McMaster University, Hamilton, ON, Canada; ^3^ Department of Oncology, Faculty of Health Sciences, McMaster University, Hamilton, ON, Canada; ^4^ Escarpment Cancer Research Institute, McMaster University, Hamilton Health Sciences, Hamilton, ON, Canada

**Keywords:** acute myeloid leukemia (AML), lysine specific demethylase 1 (LSD1), hematopoietic stem cell (HSC), leukemic stem cell (LSC), transcription factors

## Abstract

Acute Myeloid Leukemia (AML) is a type of cancer of the blood system that is characterized by an accumulation of immature hematopoietic cells in the bone marrow and blood. Its pathogenesis is characterized by an increase in self-renewal and block in differentiation in hematopoietic stem and progenitor cells. Underlying its pathogenesis is the acquisition of mutations in these cells. As there are many different mutations found in AML that can occur in different combinations the disease is very heterogeneous. There has been some progress in the treatment of AML through the introduction of targeted therapies and a broader application of the stem cell transplantation in its treatment. However, many mutations found in AML are still lacking defined interventions. These are in particular mutations and dysregulation in important myeloid transcription factors and epigenetic regulators that also play a crucial role in normal hematopoietic differentiation. While a direct targeting of the partial loss-of-function or change in function observed in these factors is very difficult to imagine, recent data suggests that the inhibition of LSD1, an important epigenetic regulator, can modulate interactions in the network of myeloid transcription factors and restore differentiation in AML. Interestingly, the impact of LSD1 inhibition in this regard is quite different between normal and malignant hematopoiesis. The effect of LSD1 inhibition involves transcription factors that directly interact with LSD1 such as GFI1 and GFI1B, but also transcription factors that bind to enhancers that are modulated by LSD1 such as PU.1 and C/EBPα as well as transcription factors that are regulated downstream of LSD1 such as IRF8. In this review, we are summarizing the current literature on the impact of LSD1 modulation in normal and malignant hematopoietic cells and the current knowledge how the involved transcription factor networks are altered. We are also exploring how these modulation of transcription factors play into the rational selection of combination partners with LSD1 inhibitors, which is an intense area of clinical investigation.

## Introduction

**Figure 1:**
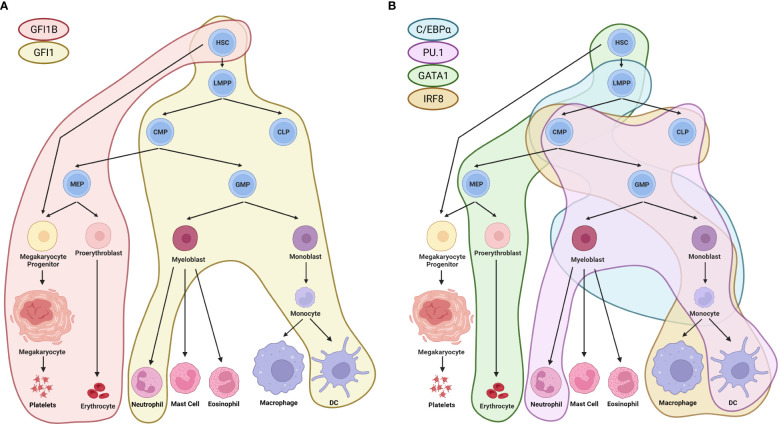
Regulation of hematopoiesis by TFs. **(A)** GFI1 and GFI1B, TFs that directly interact with LSD1, have distinct roles in the differentiation of HSCs towards different lineages. GFI1 is involved in the differentiation of HSCs towards the erythroid lineage, leading to the formation of platelets and erythrocytes (indicated by the red area). GFI1B, on the other hand, has been shown to influence the differentiation of HSCs towards lympho-myeloid primed progenitors, leading to in the formation of terminally differentiated neutrophils and dendritic cells (indicated by the yellow area) (2). **(B)** Other TFs, such as C/EBPα, PU.1, GATA1, and IRF8, which are modulated by LSD1, also play important roles in the regulation of hematopoiesis. C/EBPα regulates the differentiation trajectory of lympho-myeloid primed progenitors towards myeloblasts and monocytes (indicated by the blue area) (3). PU.1 influences the differentiation of common lymphoid and myeloid progenitors towards neutrophils and dendritic cells (indicated by the pink area). GATA1 regulates the differentiation of HSCs towards the erythroid lineage and erythrocytes (indicated by the green area) (4, 5). IRF8 controls the differentiation of common lymphoid and myeloid progenitors towards monocytes, macrophages, and dendritic cells (indicated by the beige area) (6). HSC, hematopoietic stem cells; LMPP, lympho-myeloid primed progenitors; CMP, common myeloid progenitors; CLP, common lymphoid progenitors; GMP, granulocyte-monocyte progenitors; MEP, megakaryocyte-erythrocyte progenitors. Created with BioRender.com.

In adults, hematopoietic stem cells (HSCs) are at the top of a hierarchy of progenitor cells with increasingly restricted differentiation potential that can give rise to the entire blood system. These HSCs that generate mature cell populations have two important characteristics that allow them to supply the body through the entire lifespan of an adult mammal: self-renewal and the ability to differentiate (1). The cell fate decisions that allow for the formation of the hematopoietic hierarchy through the differentiation process are tightly controlled by internal factors such as transcription factors (TFs) (**Figure 1**) and chromatin accessibility as well as external factors such as the microenvironment and cytokines. However, this process can become dysregulated due to aging and the acquisition of mutations, which can lead to the development of hematological malignancies.

Acute myeloid leukemia (AML) is a form of cancer that is characterized by an increase in proliferation and a block in differentiation leading to outgrowth of immature, undifferentiated myeloid blast cells. These blasts build up in the bone marrow, overcrowding the niche, which affects the formation of normal cells such as white blood cells, red blood cells and platelets. AML is diagnosed when more than 10-20% blasts are found in the bone marrow (7, 8). It is the most common type of acute leukemia in adults, with a median age at diagnosis of 68. AML is an aggressive cancer, and most patients with AML unfortunately die from their disease. Through next-generation sequencing, multiple mutations could be identified in AML which showed that this disease exhibits a great genetic heterogeneity (9, 10). In addition to this genetic heterogeneity, it has also been shown that there is also an organizational hierarchy in AML cells with a leukemic stem cell (LSC) at the apex of the hierarchy. Similar to normal HSCs, this cell is also characterized by the ability to self-renew and give rise to the hierarchy of leukemic cells when transplanted into immunodeficient mice (11, 12). This property of engrafting immunodeficient mice could be connected to a specific transcriptional profile (13), which has been shown to be an important predictor for prognosis. While many mutations that lead to the development of AML have been identified, most of them unfortunately still lack a therapeutic intervention. While some mutations such as in the tyrosine kinase FLT3 can be targeted with specific inhibitors (14), mutations in transcription factors as well as global epigenetic regulators are difficult to target. Furthermore, mutations found in AML can lead to a dysregulation of transcription factors such as Hox transcription factors [reviewed in (15)] as well as important myeloid transcription factors (16, 17). As a direct modulation or in particular restoration of transcription factors which have lost or altered function in AML is difficult to imagine, we are exploring in this review if epigenetic modulators could serve this role.

**Figure 2:**
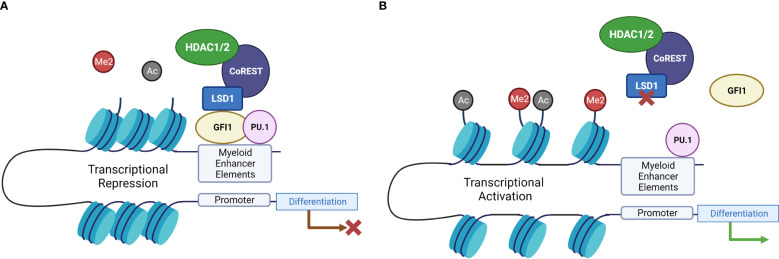
LSD1 inhibition drives differentiation-related gene expression through disruption of GFI1-mediated transcriptional repression. **(A)** When LSD1 interacts with GFI1, it recruits repressors to chromatin and catalyzes H3K4 demethylation, and histone deacetylation *via* HDAC activity, resulting in transcriptional repression. **(B)** When LSD1 is inhibited, this interaction is disrupted, leading to the displacement of LSD1 from GFI1, which in turn disrupts the repressor complex and leads to activation of enhancers and transcriptional activation of differentiation-related genes (19). Created with BioRender.com.

One candidate epigenetic regulator that has been shown to be particularly relevant in AML is Lysine specific histone demethylase 1 (LSD1 or KDM1A). It has been demonstrated to be a central epigenetic regulator in the homeostatic regulation of normal as well as malignant hematopoiesis. LSD1 is a histone demethylating enzyme specific for demethylating H3K4me1/2 or H3K9me1/2. Demethylation of H3K4me1/2 or H3K9me1/2 epigenetic marks results in transcriptional repression (at K4) or activation (at K9), however there are also non-histone targets that LSD1 is known to associate with such as the important tumor suppressor p53 (18). Chromatin remodeling can be achieved when LSD1 forms protein complexes such as the REST corepressor (CoREST) transcription repressor complex or the Mi-2/nucleosome remodeling and deacetylase (NuRD) complex, both of which contain additional epigenetic modifiers, e. g. histone deacetylase 1/2 (HDAC1/2). The epigenetic modifications resulting from histone deacetylation achieved by HDAC1/2, and the demethylation of H3K4 *via* LSD1 result in a closed chromatin conformation (heterochromatin) of relevant enhancers and therefore decreased gene transcription (18) (**Figure 2**).

**Figure 3:**
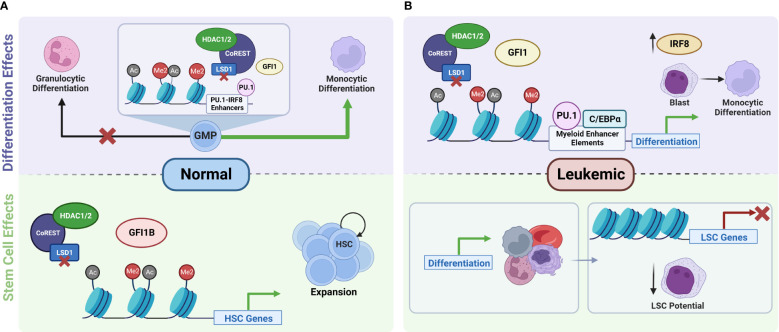
Role of LSD1 in the regulation of normal versus leukemic stem cell function and hematopoiesis. **(A)** In normal hematopoiesis, LSD1 is a key regulator of stem cell function. Inhibition of LSD1 disrupts its interactions with GFI1B, which leads to the disruption of the repressor complex and the transcriptional activation of genes responsible for the maintenance and proliferation of HSCs. This results in the expansion of hematopoietic stem and progenitor cells (23, 24, 26). LSD1 also serves as a checkpoint for cell fate decisions during the differentiation of GMPs towards granulocytic and monocytic differentiation trajectories. Knockdown of LSD1 disrupts the balance of GMP differentiation and shifts it towards monocytic differentiation (27). **(B)** In the leukemic state, inhibition of LSD1 leads to increased activity of factors at myeloid enhancer sites, leading to increased expression of downstream transcription factors such as IRF8, which promotes the differentiation of AML cells towards more mature cell types. In addition to promoting differentiation, LSD1i also leads to transcriptional repression of genes that are important for the maintenance of LSCs. Both of these effects together ultimately lead to decreased self-renewal and proliferation potential of LSCs. IRF8 gets upregulated after LSD1 knockout and accounts monocyte-biased differentiation in some AML cells (19, 28, 29). HSC, hematopoietic stem cells; GMP, granulocyte-monocyte progenitors; LSC, leukemic stem cells. Created with BioRender.com.

Previous research has determined LSD1 was expressed in 46.7% of AML specimens, compared to 6.7% in normal bone marrow indicating that LSD1 may support malignant transformation (20). The role of LSD1 in AML has been evaluated and it was found that both pharmacological and genetic ablation of LSD1 is one of the few interventions ever shown to reduce leukemic stem cell (LSC) potential and AML cell engraftment in mice (21, 22). Furthermore, LSD1 inhibition (LSD1i) leads to myeloid differentiation in leukemic cells (22). Interestingly, the effect of LSD1i on normal hematopoiesis is very different. LSD1 knock down (KD) *in vivo* results in increased HSC and progenitor potential, and transcriptional repression mediated by LSD1 is required for HSC maturation (23, 24). *In vitro*, LSD1i has been shown to expand HSC and the activity of the so far most potent mediator of HSC expansion identified UM171 has also been connected to LSD1 function (25, 26). So, while LSD1i interferes with self-renewal and induces differentiation in AML cells, it can have the opposite effect in normal HSC, where it increases self-renewal and reversibly interferes with differentiation (**Figure 3**). This has provided the impetus to develop clinically relevant LSD1 inhibitors. Many of these inhibitors are derived from tranylcypromine (TCP) that has been shown to irreversibly inhibit LSD1 by covalently binding to its co-enzyme FAD. Tranylcypromine (in combination with ATRA) has been evaluated in the treatment of AML in several clinical trials with some indication of clinical activity (30–32). However, given the lack of specificity and side effect profile that makes it difficult to achieve concentrations effective against LSD1 with tranylcypromine *in vivo* more specific and more potent inhibitors of LSD1 have been developed such as Iadademstat (ORY-1001) and Bomedemstat (IMG-7289) that are both under intense clinical investigation. There is an increasing understanding that LSD1 inhibition acts by modulating the activity of myeloid transcription factor networks involved leukemogenesis. In this review, we have therefore summarized the current understanding of these mechanisms.

## Transcription factors directly interacting with LSD1

### GFI1 and GFI1B

LSD1 has been shown to directly interact with both GFI1 and GFI1B. Both of these transcription factors play an important role in cell fate decisions that are important in the maintenance of the hematopoietic system. GFI1 and GFI1B are nuclear zinc finger proteins that mostly act as transcriptional repressors. GFI1 and GFI1B also have an auto-regulatory ability, where they can bind to their own promoter region and either activate or repress transcription. This phenomenon of negative auto-regulation has been observed with both GFI1 and GFI1B (33, 34). This regulatory feedback pathway also involves the interplay with lineage specific transcription factors. For example, it has been suggested that GFI1B can self-regulate through interacting with GATA1, a key factor involved in erythroid differentiation. GATA1 can bind to GATA and GFI1 (AATC) sites to activate GFI1B transcription, and GFI1B expression is inhibited by negative regulation of Gfi1b through protein complex formation between GATA1 and the SNAG domain of GFI1B (33). Furthermore, GFI1/1B cross-regulation is possible because they share the same DNA-binding motifs. This has been shown *in vivo* where when GFI1 is knocked-in, it can repress transcription from a GFI1B promoter reporter (34). GFI1 and GFI1B exhibit their repressor function by binding directly to DNA and by recruiting LSD1 also recruit corepressor complexes including histone deacetylases [reviewed in (2)]. LSD1 plays a critical role for this scaffolding function, as it directly interacts with GFI1 and GFI1B, which could be shown by co-immunoprecipitation as well as by functional experiments in which knockdown (KD) of LSD1 resulted in a decreased level of LSD1 and CoREST associated with GFI1B, indicating that LSD1 mediates the formation this protein complex (28, 35). LSD1/CoREST/HDAC2 are recruited by the SNAG domain of both GFI1b and GFI1 and are tethered to the majority of gene targets *in vivo* (35). The SNAG domain is conserved between both GFI1 and GFI1B and when it is mutated (SNAG-P2A mutation) these proteins lose their functional ability to promote differentiation, indicating that this domain is critical to the functional interaction with LSD1 and the formation of the CoREST complex (35). Therefore, GFI protein complexes can catalyze histone modifications of their target genes *via* actions of LSD1 and CoREST, ultimately leading to gene silencing which is required in lineage choice and thereby modulates hematopoietic differentiation (24, 35).

In the hematopoietic system the two factors exhibit an interesting dichotomy. In HSCs, GFI1B expression is increased and GFI1 is repressed, but when these cells begin to differentiate into multi-potent progenitors (MPPs), the opposite is true – GFI1B expression is decreased and GFI1 becomes upregulated (36) (**Figure 1**). Furthermore, GFI1 is expressed in the granulocyte/monocyte and lymphoid lineage, whereas GFI1B is absent in these cells. GFI1B is most highly expressed in megakaryocyte-erythrocyte progenitors and during maturation of these cell types [**Figure 1**, reviewed by (36)].

Based on its expression pattern GFI1 plays an important role in granulocytic differentiation and germline mutations in GFI1 are associated with severe congenital neutropenia (37). It has also been shown that a variant allele of GFI1 has been found associated with the development of AML (38). A knockdown model of GFI1 accelerated the development of several leukemia models and led to the development of a fatal myeloproliferation (39), however not a complete knockout. In addition to its role in differentiation Gfi1 also has an important role in preventing and regulating apoptosis by interacting with p53 which has been shown in T cells (40), ALL (41) and as mentioned above also in myeloid cells (39). A complete loss of Gfi1 therefore led to an activation of p53 and the subsequent induction of apoptosis. This activation of p53 is associated with an increase in Lysine 372 methylated p53. Interestingly, it has also been shown that the effect of LSD1 inhibition also depends on the effect of p53 as demonstrated by Cai et al. in elegant studies comparing the effect of LSD1 inhibition in LSK- and GMP-derived MLL-AF9 driven murine leukemias (42). This effect could in part be overcome by cotreatment of AML cells with LSD1 inhibitor and Venetoclax.

As mentioned above the interaction of GFI1 with LSD1 and the CoREST is important for its regulatory role in hematopoietic differentiation. In line with this, when AML cell lines are treated with an LSD1 inhibitor, transdifferentiation is observed (43). After treatment, there was significant enrichment for PU.1 binding site gene sets which indicates that the treatment triggers aberrant upregulation of myeloid lineage genes. Specifically, LSD1i caused monocytic lineage gene signature upregulation and downregulated the erythroid gene signature (43). A megakaryocytic AML cell line that was treated lost its characteristic gene signatures and acquired both natural killer cell and monocyte lineage gene signatures (43). There were similar findings by others where treatment of MDS-related AML cells or the AML cell line THP1 with different covalent LSD1 inhibitors induced upregulation of genes associated with myeloid differentiation such as GFI1, CEBPA, PU.1, IRF8, and the leukemic stem cell gene signature was repressed (19, 44). An upregulation of the myeloid transcription factors GFI1, PU.1, and C/EBPα was also observed at the protein level, whereas CoREST and DNMT were reduced after both pharmacological LSD1i and LSD1 knockout in AML cells (44, 45). The same trend was observed with cell surface marker expression as granulocyte/macrophage markers CD86 and CD11b were upregulated after treatment, whereas erythroid markers (43, 44) and the stem cell marker cKit were decreased (19, 28) (**Figure 3**). When murine AML models are subjected to conditional LSD1 knock out, the same myeloid differentiated immunophenotype is observed, however these cells also gain expression of F4/80, stem cell marker Sca1, and FcεRI, which differs from normal physiological differentiation markers and indicated an atypical differentiation with both features of granulocytic as well as monocytic differentiation (28). This aberrant differentiation is understandable as LSD1 mediated repressive effects are probably required for faithful lineage choice. *In silico* transcription factor motif prediction revealed that GFI motifs were among the most strongly activated motifs after LSD1 knockout.

As discussed previously GFI1 can directly interact with LSD1, therefore it was hypothesized that activation of genes repressed by GFI1 may facilitate the effect of LSD1 inhibition (28, 35). Immunoprecipitation of endogenous GFI1 in AML cells pulls down LSD1 and LSD1 inhibition with covalent inhibitors disrupts this interaction (19). However, LSD1i does not interfere with the interaction between LSD1 and other CoREST complex members such as RCOR1 or HDAC1/2. The interplay between enzymatic versus scaffolding functions of LSD1 has also been investigated in the context of AML. Research using cells with an enzymatically inactive mutant form of LSD1 still showed activity of additional treatment with a covalent LSD1 inhibitor. While some differentiation and upregulation of Gfi1b were still observed when an enzymatically inactive mutant was induced, the interaction of the LSD1 mutant and GFI1 is still reduced by LSD1 inhibition as observed with immunoprecipitation (19, 28, 46). However, *in vivo* the enzymatically inactive LSD1 was not able to produce a survival benefit as compared to LSD1 knockout (28). Furthermore, another LSD1 mutant that is unable to bind with GFI1 was generated in AML cell line MOLM-13 that are resistant to ATRA. When wild type LSD1 is knocked out in these cells, differentiation is potentiated after treatment with retinoic acid. When WT LSD1 or the enzymatically inactive mutant LSD1 are added back into the cells, the WT phenotype is restored, however when the scaffolding mutant (unable to bind with GFI1) is added to the cells, the phenotype is not restored, and the cells show enhanced sensitivity to retinoic acid (46). Additionally, when GFI1 is knocked down in OCI-AML5 cells, and treated with an LSD1 inhibitor, the expression of the differentiation markers CD11b and CD86 are enhanced (45). These results indicate that the LSD1-GFI1 interaction is crucial for the differentiation block characteristic of AML, and both the scaffolding and enzymatic functions of LSD1 need to be targeted to retain the differentiative and anti-leukemic activity of LSD1 inhibition. Interestingly, the effect of LSD1 inhibitors on the scaffolding function of LSD1 may be limited mostly to the covalently acting tranylcypromine derivatives which makes them ideal as drug candidates for the treatment of AML (28).

The mechanism of LSD1 inhibition in AML may also involve its activity on super enhancers. Super enhancer activation of LSD1’s direct targets such as GFI1 by LSD1 inhibition is an initial event occurring before gene upregulation and myeloid differentiation that is observed in leukemia (44). It was observed that after LSD1i treatment, H3K27 acetylation at the GFI1 super enhancer was elevated after 3 hours, the transcript level of GFI1 increased after about 12 hours, where CD11b was induced after 24 hours of treatment. This is indicating that the GFI1 super enhancer is activated by LSD1 inhibition as a first step before the induction of myeloid gene transcription and differentiation (44). Furthermore, when GFI1 is depleted, the myeloid differentiation induced by LSD1 inhibition is weakened, indicating that GFI1 is a target of LSD1 in leukemia (44). Silencing of GFI1 by suppression of the GFI1 super enhancer by LSD1 could be one of the features of the myeloid differentiation block found in AML.

GFI1B also has important roles in normal hematopoiesis and leukemogenesis. Mutations in GFI1B cause a deficiency in platelet number and function (47). In the context of leukemia models deficiency of Gfi1b accelerates leukemia development both in a haploinsufficient, but also full knockout model (48). GFI1B can also have differential effects on lineage commitment in the leukemic and in the normal context. In human primary progenitor cells, the expression of GFI1B is increased during erythroid maturation, and when GFI1B is knocked down terminal differentiation is delayed. However, it has the opposite role in leukemic cells. Forced expression of Gfi1b in leukemic cell lines leads to an induction of differentiation along the erythroid lineage. This indicates that GFI1B affects late-stage erythroid differentiation as a transcriptional repressor both in normal and leukemic progenitor cells (49). LSD1 inhibition can also disrupt the association between LSD1 and GFI1B in a concentration-dependent manner (43). The LSD1-GFI1B complex represses the GFI1B promoter and therefore LSD1 knockdown leads to a subsequent increase in GFI1B expression in both normal as well as leukemic models (28, 43). Conditional LSD1 KD in mice results in significant expansion of the HSC and progenitor compartment, specifically enhanced in the LSK CD48^-^ CD150^+^ population as assessed by flow cytometry. Similar to what was found by Saleque et al. there was four times more de-repression of GFI1B in the KD condition versus control, indicating that loss of LSD1 *in vivo* causes overexpression of GFI1B due to its derepression (24). However, as the activity of GFI1B also requires the recruitment of LSD1, it is unclear what the functional role of this upregulation is for the biological phenomena associated with LSD1 inhibition such as normal HSC expansion or induction in differentiation of leukemic cells. Some indications in that regard come from data showing that when LSD1 and GFI1B are both knocked down, this upregulated CD86 and reduced proliferation in an additive manner. These findings support the idea that the disruption of LSD1-GFI1B complexes can also enhance differentiation in AML down a myeloid lineage (19, 43) (**Figure 2**).

On the level of the hematopoietic stem cell, Gfi1b has been shown maintain quiescence and a loss of GFI1B was shown to result in increased metabolic activation in HSCs. The Gfi1b-deficient HSCs showed an increased level of reactive oxygen species (ROS) compared to wild type HSCs and treatment with N-acetylcysteine (NAC), which counteracts ROS, significantly limited the expansion of HSCs, suggesting that ROS may play a critical role in Gfi1b-deficient HSCs expansion (38). This indicates that Gfi1b may play a role in regulating the metabolism and ROS levels of HSCs, and that altered Gfi1b expression or activity may affect the expansion and function of these cells. Recently, Gfi1b has been shown to regulate oxidative phosphorylation (OxPhos), fatty acid oxidation (FAO), and c-Myc expression in hematopoiesis and leukemogenesis. Gfi1b deletion significantly activated OxPhos and altered the energy metabolism of cells to rely more on oxidative phosphorylation, causing cells to shift their dependency on glucose for energy to using fatty acids through the upregulation of FAO. The progression from preleukemia to leukemia was accompanied by changes in the metabolic phenotype and interestingly, genetic variations in AML cells were found to have a great influence on the correlation between Gfi1b expression and the metabolic phenotype (50). This suggests that the role of Gfi1b in regulating energy metabolism in hematopoietic and leukemic cells may be dynamic and may vary depending on the specific stage of disease progression and the genetic characteristics of the AML.

It is tempting to speculate that the differential effect of LSD1 inhibition in normal and malignant hematopoiesis may be mediated by differential interference with the effect of GFI1B or GFI1. Whereas interfering with GFI1B may mediate the stem cell expansion observed with LSD1 inhibition in normal HSC, interfering with GFI1 function could be responsible for many of the observed effects on AML cells.

### SCL/TAL

Stem Cell Leukemia/T-cell acute leukemia 1 (SCL/TAL1) is another transcription factor that interacts directly with LSD1. It has been shown to be essential for primitive hematopoiesis, and populations with erythroid differentiation potential (51). SCL/TAL1 is silenced by PU.1 binding to its silencer region in erythro-megakaryocytic cells (51). Furthermore, it has been shown that SCL/TAL1 associates with core members of the LSD1-CoREST complex such as HDAC1/2, which is required for SCL/TAL1 mediated transcriptional repression, in both T cell leukemia and normal erythroid progenitor cells (52). Knockdown of LSD1 in differentiated murine erythroleukemia cells or embryonic stem cells induces GATA2 and decreases GATA1 expression. It was subsequently shown that LSD1 is recruited to the GATA2 locus *via* interactions with SCL-TAL1, leading to epigenetic silencing of GATA2 (53). As mentioned earlier, GATA1 is a key factor in differentiated cells, while GATA-2 is highly expressed in stem cell populations. This interaction between SCL/TAL1 and LSD1 is therefore involved in target gene regulation responsible for erythroid development (52). Much less is known about the role of SCL/TAL1 in the context of AML.

## Transcription factors modulated by LSD1

While LSD1 directly interacts with some transcription factors many more get modulated indirectly in their activity either because LSD1 regulates the accessibility of enhancers targeted by these transcription factors or because LSD1 is directly involved in the regulation of the transcription factor. In the following part we will therefore first summarize the impact of LSD1 inhibition in this regard.

As LSD1 is recruited to myeloid enhancer regions through the effect of GFI1/1B, it can regulate the activity of transcription factors that typically bind to myeloid enhancers (**Figure 2**). The two most relevant in this context are PU.1 and CEBPA. One transcription factor directly regulated by LSD1 is IRF8, an important monocyte differentiation factor (**Figure 2**).

### PU.1

HSCs express PU.1 (Spi-1) and knock-out models have shown that its constitutive expression is necessary for HSC maintenance, as PU.1 knock out animals develop a differentiation block (54). PU.1 is also a very important regulator in the development of the myeloid lineages, granulocytes, and monocytes. It is highly expressed in the Granulocyte Monocyte Progenitor (GMP) population and expressed in the majority of HSCs that are capable of self-renewal. PU.1 is capable of regulating fate choice by its graded expression. For example, high concentration induces macrophage differentiation and low concentration of PU.1 induces B-cell differentiation, however PU.1 is not necessary for B-cell maturation (54, 55). Furthermore, PU.1 is involved with regulating multiple factors involved in regulation of HSC cell division. HSCs with decreased PU.1 levels are dysfunctional and cannot regenerate bone marrow in competitive repopulation or serial transplantation assays (56). HSCs with decreased PU.1 have an increased actively dividing population compared to wild type cells which indicates that PU.1 directly regulates proliferation, and downstream target genes that affect proliferation such as Wnt, MAPK, and P53 are significantly enriched (56). Furthermore, decreased PU.1 levels result in decreased expression of negative regulators of the cell cycle such as the transcription factor Gfi1 (discussed in detail above) or the cyclin-dependent kinase inhibitor Cdkn1a (p21), whose transcription is directly regulated by PU.1. Similarly, decreased PU.1 levels result in an enhanced expression of cell cycle activators, and PU.1 negatively regulates their transcription (56). The maintenance of normal PU.1 levels in HSCs is achieved in an autoregulatory fashion, which is what balances the activity of the cell cycle inhibitors and activators (56). Therefore, the function of PU.1 as a cell-cycle regulator, when balanced by autoregulation, can control and maintain HSC growth and self-renewal, and when PU.1 expression becomes unbalanced it leads to changes in expression of both cell cycle activators and inhibitors ultimately leading to proliferation (and stem cell exhaustion) in HSCs.

Interestingly, PU.1 is also involved in feedback regulation cycles with other hematopoietic regulators such as GFI1. It was found that these two factors mutually inhibit each other which leads to the development of different differentiation programs. For instance, lymphoid factor Ikaros induces GFI1 expression which promotes B cell differentiation by antagonizing the expression of PU.1, and therefore myeloid differentiation programs in progenitor cells (57). Progenitor cells initially express high levels of PU.1, and cells continuing to express it develop macrophage immunophenotype (CD11b/F4/80), whereas cells that begin to downregulate PU.1 express B cell marker (CD19) (55, 58). It appears that the modulation/autoregulation of PU.1 levels in these cells is achieved through cell cycle modulation, for example, increasing cell cycle length allows for higher PU.1 levels to be maintained, as is the case for macrophages (58). PU.1 is involved in the myeloid/lymphoid cell fate choice *via* interactions with myocyte enhancer factor 2c (MEF2c), which is crucial for lymphoid development from the MPP stage. MEF2c acts downstream of PU.1 and is directly regulated by it (59).

Just as the graded expression of PU.1 can dictate lineage decisions during normal hematopoiesis, this function is also involved in leukemogenesis. A graded reduction of PU.1 expression, rather than a complete loss, can induce AML. For instance, if PU.1 is downregulated to about 20% of its normal activity (either by knockdown or modulating an important enhancer) this leads to the development of AML (60). PU.1 knock down animals develop hypercellularity of the liver and spleen due to expansion of myeloblasts, and greatly accumulate immature myeloid cells (cKit^+^ Mac-1^low^ GR-1^low^) (60). This could be due to the fact that at a 20% reduction of PU.1, the cells are able to retain their ability to respond to cytokines essential for cell survival. Furthermore, in both AML cell lines and patient samples, transduction with PU.1 leads to increased apoptosis, and artificially increasing the PU.1 levels in these cells influences them towards myelomonocytic immunophenotype, differentiation into macrophages, and decreased proliferation correlated with diminished S-phase cell cycle kinetics (61). These changes all correspond to a shift from immature myeloid blast-type phenotype towards a mature, terminally differentiated, and non-proliferating myeloid cell type (61). All together this lends to the idea that small modulations in key transcription factors and their downstream networks can change cancerous cell fate (60).

It appears that modulation of TF networks involving PU.1 are central to the effects of LSD1 inhibition in AML. For example, myeloid transcription factor networks are strongly activated upon LSD1 knock out in H9M and MN1 murine leukemia cells (28). As mentioned previously, transcription factor motif analysis indicated that the most strongly activated TFs were predicted to be those involved with myeloid differentiation, namely PU.1, Cebp, Gfi1/1b. Further RNA-seq analyses indicated that even though these motifs were predicted to be active, the expression level was not changed. This lends to the idea that GFI1 may be acting as a repressor on PU.1 target genes (28). In the context of MLL-AF9 AML, treatment with LSD1 inhibitors induces gains in chromatin accessibility with enrichment of myeloid TF motifs for PU.1, C/EBPα-b, and RUNX1 (29). Chip-seq studies to identify sites with occupancy of these TFs after LSD1 inhibition revealed that there was a predominantly global gain in PU.1 occupancy after treatment, and a loss of C/EBPα signal, leading to a model where these sites have pre-existing occupation of major myeloid differentiation factors, but their activity is inhibited by the activity of LSD1 (29). Pharmacological LSD1 inhibition hinders the interaction between LSD1 and GFI1, and triggers transcription of key myeloid genes including IRF8, KLF4 and MEF2C *via* histone acetylation (19). Therefore, antagonistic activity between PU.1 and other factors, such as GFI1, appear to be central to the consequences of LSD1 inhibition in AML, likely due to the interference of GFI1-mediated repression of PU.1 target genes (19, 28). This shows that targeting the transcriptional programs involved in certain subtypes of AML with use of epigenetic modulators such LSD1 inhibitors appears promising.

### C/EBPα

Another relevant transcription factor in the development of AML is CCAAT/enhancer binding proteins (C/EBP) α. There is evidence that C/EBPα is recurrently mutated in AML, and even WT C/EBPα is a key factor in the differentiation program required for AML initiation (62). A knock-in of mutant C/EBPα can induce AML in a murine model (63). It is also relevant in core binding factor (CBF) translocated AML, which share epigenetic similarities to C/EBPα mutated AML and in which C/EBPα is found downregulated (16).

C/EBPs are central factors to granulopoiesis and are also involved in cell proliferation. In purified stem and progenitor cells C/EBPα is expressed in low levels in HSCs but then is upregulated upon differentiation to CMPs and GMPs, it is not present in MEPs or lymphoid cells (64). Furthermore, when C/EBPα is abrogated the transition from CMP to GMP is blocked and results in an accumulation of blasts sharing similarities with human AML (64). The activity of C/EBPs is involved in limiting stem cell self-renewal, as C/EBPα KO in HSCs increased levels of polycomb complex gene Bmi-1, leading to a competitive advantage over WT HSCs (64).

On the other hand, C/EBPα is also required during leukemogenesis. HOXA9 is a HOX group transcription factor that has an important part in development and hematopoiesis, consequently it is highly expressed in HSCs and early progenitors and promotes their proliferation, therefore upon differentiation it is downregulated. However, HOXA9 is very frequently overexpressed in AML and correlates with poor prognosis (65). C/EBPα was identified as a key collaborator that is enriched at HOXA9 binding sites, and is critical for maintaining proliferation *in vitro*, and directly contributes to severity of Hoxa9-driven leukemia *in vivo* (65). In addition, MLL-fusion proteins are inducers of leukemogenesis that depend on HOXA9/MEIS1 for their oncogenic transcriptional programs (66). It was found that C/EBPα is required for MLL-ENL dependent AML and when deleted, MLL-ENL transduced HSPCs did not give rise to leukemia or any dysfunctional hematopoiesis when transplanted into mice. However, when Cebpα is ablated in already existing leukemia *in vivo*, it does not change the overall survival or immunophenotype of these animals, this indicates that C/EBPα is not required for maintenance of MLL-ENL AML cells but has a defined role during leukemogenesis (66).

As discussed previously the interplay between the key myeloid TFs PU.1 and C/EBPα are central to the LSD1 inhibition effect. For example, LSD1 inhibition in OCI-AML3 cells induces protein level expression of C/EBPα and other factors such as p53 and p21. This corresponds to an induction of differentiation with observed morphologic features of differentiation, and the myelo-monocytic marker CD11b is upregulated in OCI-AML3 and primary patient specimens *in vitro* (67). In MLL-AF9 AML, after treatment with an LSD1 inhibitor, there was a global gain in open chromatin occupancy of PU.1, but not of C/EBPα (29). However, when C/EBPα or PU.1 is knocked out in MLL-AF9 AML cells, the increased chromatin accessibility is diminished which indicates that both of these factors are necessary for changes to chromatin conformation after LSD1 inhibition, and when either factor is deleted, this confers resistance to LSD1 inhibition in this context (29).

As mentioned previously, C/EBPα is often mutated in AML, and these mutants typically co-occur with mutations in the granulocyte colony stimulating factor receptor (CSF3R). These mutations cause ligand-independent activation of the downstream JAK/STAT pathway, ultimately causing neutrophilic leukemia. When both are mutated, it eventually leads to a differentiation block downstream of CSF3R, causing a highly aggressive form of AML (68). It was found that LSD1 inhibition is highly active in CEBPA/CSF3R double-mutant AML, as well as JAK/STAT inhibitors. RNA-seq studies done on double-mutant AML cells treated with LSD1 inhibition showed that there was significant upregulation of genes associated with myeloid maturation, namely GFI1/1b, and genes associated with neutrophil activation and immune responses (68).

C/EBPα deficiency has been shown to induce metabolic reprogramming in AML, which may be associated with differentiation blockade and the development of malignant phenotypes in these cells. Specifically, LSD1 has been shown to facilitate the function of the erythroid transcription factor GATA1, while repressing the granulo-monocytic transcription factor C/EBPα. This results in dominance of GATA1 over C/EBPα, leading to the expression of metabolic genes that are specific to erythroid leukemia (EL) cells, such as those involved in glycolysis and heme synthesis (69).

### IRF8

Interferon consensus sequence binding protein (ICSBP), also known as interferon regulatory factor 8 (IRF8) is a member of the IRF transcription factor family. These TFs contain a DNA binding domain that is contained in many downstream target genes in the interferon-stimulated response element (ISRE), and associated with other factors, such as PU.1, that have an Ets-IRF composite element (EICE) (70). Through interactions with crucial binding partners, IRF8 heterodimers are able to act as either a transcriptional activator or repressor to modulate gene expression that is essential for the differentiation of monocytes such as macrophages and dendritic cells (70–72). Moreover, IRF8 is required for Ly6C+ monocyte differentiation through activating downstream target Krüppel-like factor-4 (KLF4) (71). IRF8 inhibits granulocytic differentiation from myeloid progenitor cells, which involves the repression of genes that participate in granulocytic differentiation such as Cebpa and Cebpe (70). Furthermore, in progenitor cells IRF8 interacts directly with and inhibits C/EBPα, leading to inhibition of chromatin binding by C/EBPα and ultimately inhibits neutrophil differentiation (72). This indicates that IRF8 is important for regulating differentiation by acting as both a transcriptional repressor and an activator.

The actions of transcription factors, such as IRF8, in different progenitor states influence cell fate choice. For example, single cell sequencing experiments have revealed that rare subpopulations of intermediate cell types that downregulate the stem/progenitor gene programme but have low level expression of myeloid TFs like IRF8 and GFI1, which may ultimately dictate their terminal differentiation (27). GFI1 is required for normal granulopoiesis, and IRF8 is necessary for monopoiesis (as mentioned above), and when either IRF8 or GFI1 is removed in GMP cells, it triggered disruption of downstream TFs associated with monocytes and granulocytes, respectively. IRF8 is a target of GFI1 antagonism of the monocytic-dendritic differentiation programme in GMP cells by repressing enhancers activated by the PU.1-IRF8 heterodimer (27). Furthermore, IRF8 can trigger distinct differentiation programs by establishing enhancer landscapes in progenitor populations by its binding interactions with PU.1 and antagonism of C/EBPs. For instance, it has been proposed that lineage choice is determined by IRF8’s interactions with enhancers and other TFs in a concentration-dependent manner (73). High IRF8 expression created dendritic cell lineage enhancers by formation of heterodimers IRF8-PU.1 on chromatin which inhibits C/EBP binding. However low IRF8 expression creates monocyte-lineage enhancers in a similar way, except binding of C/EBP is allowed. In the absence of IRF8 neutrophilic enhancers are created by C/EBP and PU.1 (73). These studies indicate how the actions of IRF8 on enhancer regions of lineage-determining genes can dictate terminal differentiation (**Figure 3**).

IRF8 has been identified as a tumor suppressor in AML, and when IRF8 is removed from mice the animals develop a spontaneous disease with similarities to human CML, including a transition to blast crisis in about a third of animals (74, 75). The bone marrow of IRF8 deficient mice had significant hypercellularity with a distinct increase in mature granulocytes, further lending to the role of IRF8 in suppressing granulocytic differentiation (71, 75). This indicates that IRF8 is important to regulating hematopoiesis and its dysregulation can lead to oncogenesis, therefore IRF8 may serve as a biomarker in AML (76).

The meningioma 1 (MN1) murine leukemia model is an important tool for studying treatment resistant AML, as these cells are about 3000 times more resistant to all trans retinoic acid (ATRA) (77). In this model the characteristic myeloid differentiation block is achieved by transcriptional repression of target myeloid genes, such as IRF8 (78). Overexpression of IRF8 in MN1 cells leads to reduced cell cycling and increased monocytic phenotypes such as granulation and phagocytosis. Furthermore, overexpression of IRF8 *in vivo* in AML xenograft models resulted in decreased tumor volume, indicating the potential of IRF8 as a potential therapeutic target (78). It has also been observed that AML cells can gain therapeutic resistance without a new genetic mutation conferring that resistance, and it is thought that this could be due to transcriptional plasticity at enhancer sites. Furthermore, it is possible to overcome non-genetic drug resistance by treatment with LSD1 inhibitors. In the case of BET inhibition, LSD1i in drug resistant cells induces mature myeloid differentiation and appears to reinstate the ability of BET inhibitors to repress a set of genes critical for cancer survival, such as Myc and Bcl2 (79). Utilizing ATAC-seq it was possible to determine that this effect is due to the formation of new enhancers around these key survival genes, and the most enriched TF binding sites at these new regions were again the PU.1 motif and EICE motif for the IRF8-PU.1 heterodimer (79). These results indicate that targeting enhancer remodelling, instead of the final transcriptional endpoint, may be beneficial when treating resistant leukemia.

## Combination treatments with LSD1i

Although LSD1 plays a significant role in the development of leukemia and is highly expressed in AML, the efficacy of LSD1 inhibitors as a monotherapy is limited. In a study with the highly effective LSD1 inhibitors Iadademstat only one of the patients achieved a complete response even though the observed cellular responses were very impressive with major differentiation effects in many of the patients treated (80).

One of the first combination approaches pursued with LSD1 inhibition was to combine it with ATRA. As described above, even first indications of activity of LSD1 inhibition in AML were seen with this combination (22). This approach was also used in the above-mentioned clinical studies that combined LSD1 inhibition with tranylcypromine with ATRA. Concomitant pre-clinical studies using this combination in a murine Hoxa9/Meis1 (H9M) driven leukemia model and resistant MN1 cells suggest that the combination with ATRA may mostly enhance a preexisting sensitivity to ATRA as LSD1 inhibition was not able to overcome the resistance against ATRA in MN1 cells (28). Transcriptomic data showed that an enhanced activity of IRF8 was found in H9M cells, which could potentially account for the atypical monocyte-biased differentiation process induced after LSD1 KO in this model as well as the lack of ATRA sensitization in MN1 cells where this factor did not change activity (28).

Another approach is to combine LSD1 inhibition with more standard therapy. This approach is currently pursued with Iadademstat that is being tested in a clinical trial in combination with the hypomethylating agent azacytidine. This combination has yielded promising response rates (81). However, recent studies using small molecule drug screening and rational drug combination approaches provided evidence that LSD1 inhibition can sensitize AML cells also to the effects of other inhibitors that would not be active as a monotherapy. This suggests that targeting multiple pathways in combination with LSD1 inhibition could be a more effective strategy for treating AML (**Table 1**).

**Table 1:** Summary of pre-clinical findings assessing the effect of LSD1 inhibitors in combination with other targeted therapies on treatment response in AML.

Target	Combination Agent	LSD1 Inhibitor	Leukemia Subtype/Model	Treatment Response	Reference
RAR/RXR	ATRA	TCP	non-APL primary AML cells and HL60 H9M, MN1 murine models	Superior activity of combination in PDX and xenograft models. LSD1i enhances sensitivity to ATRA in H9M.	(22) (28)
mTORC1	RAD001	OG-86	THP1 AML cells and xenografted primary *MLL*-translocated AML cells	Synergistic activation of myeloid differentiation in combination treatment *in vitro* and *in vivo*.	(82)
JAK/STAT	Ruxolitinib	GSK-LSD1	CSF3R/CEBPA double mutant murine AML	Synergistic reduction in cell proliferation, increased myeloid differentiation *in vitro*, and improved OS *in vivo.*	(68)
KIT	Avaprintinib	ORY-1001	KIT-mutant AML primary patient samples and Kasumi-1	Synergistic reduction in cell proliferation *in vitro* and *ex vivo*.	(83)
HDAC	Panobinostat	SP2509	OCI-AML3, MOLM-13, primary patient samples, and PDX models	Synergistic reduction of cell proliferation in cell lines and primary AML blasts *in vitro*. Improved OS in PDX models.	(67)
EZH2	EPZ6438	SP2509	ML1, HL60, and xenograft model (HL60)	Synergistic induction of apoptosis, and reduced OxPhos after combination treatment *in vitro*. Synergistically decreased tumor volume *in vivo*.	(84)
BCL2 inhibitor	Venetoclax	Bomedemstat (IMG-7289)	MLL-AF9 AML syngeneic model	Reduction in leukemic burden and higher induction of apoptosis in combination treatment *in vivo*.	(42)
BRD4	OTX015	INCB059872	Primary AML blasts, OCI-AML5 xenograft	Combination showed synergistic lethal activity *in vitro*. Improved OS in combination treatment *in vivo*.	(45)
Nucleic Acid Metabolism Lipid Metabolism	6-mercaptopurine Cerulenin	GSK-LSD1	ER-HOXA9 murine model	Combination treatment enhanced differentiation *in vitro*.	(85)

A genome-wide CRISPR-Cas9 dropout screen identified multiple components of mTORC1 signaling as sensitizers to the treatment with LSD1 inhibition. This led the authors of this study to test a combination of mTORC1 inhibition with LSD1 inhibition in MLL-translocated AML which led to enhanced differentiation in both cell line and primary cell setting. The combination of LSD1 inhibition with mTORC1 inhibition would therefore represent one of these combination approaches (82).

When LSD1 inhibition is used in concert with a JAK/STAT inhibitor, it results in synergistic ablation of differentiation block and induces maturation of AML cells. The combination treatment also induces complete hematologic disease control and significantly increases survival *in vivo*, while treatment alone does not improve survival in CSF3R/CEBPA double mutant murine AML models (68). These findings provide a classic example of how LSD1 inhibition can be an effective treatment when used in combination with a targeted kinase inhibitor in AML.

KIT inhibitors, in concert with LSD1 inhibitors could be a potential avenue of treatment for KIT-mutant AML, as this subtype has significantly increased LSD1 expression compared to normal CD34+ samples (83). It was found that the combination of LSD1 inhibition and KIT inhibitor avaprintinib significantly decreases proliferation in KIT-mutant AML cell line Kasumi-1, and patient samples, in a synergistic fashion. RNA-sequencing revealed that cells treated with the combination had increased enrichment for PU.1 and downregulation of MYC (key regulator of growth and survival) target genes, which could explain the synergistic effects (83). Therefore, the modulation of transcription factor networks involved with differentiation and cell growth can be modulated by LSD1 inhibition in combination with targeted kinase inhibitors in specific AML subtypes.

Inhibiting histone deacetylase (HDAC) has been found to cause apoptosis, differentiation and inhibit the progression of the cell cycle in AML cells (86, 87). Combination of LSD1 inhibition with LSD1 inhibitor, SP2509, and HDAC inhibition with the pan-HDAC inhibitor, Panobinostat resulted in synergistic apoptosis in *in vitro* AML models including OCI-AML3 and MOLM-13 cell lines, and primary patient samples. The combination treatment also significantly improved the survival in OCI-AML3, and patient derived xenograft AML models (67). This suggests that the combination of LSD1 inhibition with HDAC inhibition could be an effective therapeutic strategy for treating AML.

Enhancer of zeste homolog 2 (EZH2) is a histone lysine methyltransferase that alters epigenetics by catalyzing H3K27 methylation. Despite having contrasting histone methylation functions, both EZH2 and LSD1 are elevated in AML cells. Co-inhibition of EZH2 and LSD1 using EPZ6438and SP2509, respectively, has been shown to induce epigenetic alterations coupled with aberrant Oxphos and glycolysis, resulting in the depletion of ATP, ultimately leading to synergistic induction of apoptosis in AML cells (84).

LSD1 inhibition has recently been shown to sensitize chemoresistant high-risk leukemias to the apoptosis-inducing effects of Venetoclax (42). LSD1 inhibition using bomedemstat (IMG-7289) showed significant interactions with venetoclax resulting in synergistic reduction in AML cell proliferation and increased apoptosis of AML blasts *in vitro* and reduction of leukemic burden *in vivo* (42). This suggests that the combination of LSD1i and Venetoclax could be an effective treatment strategy for high-risk leukemias which is currently under clinical investigation (NCT05597306).

A recent study using CRISPR-Cas9 screening on LSD1i treated AML cells has identified bromodomain-containing protein 4 (BRD4) as one of the co-dependencies (45). Consequently, co-treatment with a BRD4 inhibitor, OTX015, and an LSD1 inhibitor, INCB059872, has been found to induce synergistic apoptosis in various AML and post-MPN secondary AML cell lines with different genetic alterations. This combined treatment also led to improved survival and reduced leukemic burden *in vivo* (45).

A recent small molecule chemical library screen identified two compounds, 6-mercaptopurine and cerulenin, that act synergistically with LSD1i to effectively circumvent differentiation blockade and advance the maturation of AML cells (85). Treatments using a combination of LSD1i and 6-mercaptopurine, an inhibitor of purine metabolism, or LSD1i with cerulenin, an inhibitor of *de novo* fatty acid synthesis, resulted in a synergistic increase in the proportion of differentiated cells in a time- and dose-dependent manner, when compared to individual treatments in mouse AML cells. Furthermore, the greatest increase in the proportion of differentiated cells was observed when all three agents were used together (85). This shows the interactions between specific metabolic pathways and epigenetic modifications are potential druggable targets to improve differentiation block in AML.

## Conclusions

As AML could be seen as an accumulation of rapidly expanding progenitor cells in hematopoiesis that could be seen as transient stage in differentiation. As outlined above these often dependent on the activity of myeloid transcription factors such as PU.1 or C/EBPα, but on the other hand must limit their activity to sustain their differentiation block and prevent terminal differentiation. LSD1 as repressive epigenetic regulator could in this way play a role at limiting the activity of factors contributing to terminal differentiation. Blocking the effect of the repressive chromatin regulator LSD1 thereby could overcome this deregulation and help to destabilize the pathological LSC population. This then leads to the acquisition of differentiation characteristics. The resulting differentiation is accompanied by changes in cellular metabolism as well as the induction of cell death through p53-dependent mechanism which then make the cell also more susceptible to other interventions. The profound effect on LSCs makes LSD1 inhibition a particularly promising treatment for the maintenance setting. The sensitization seen to many other interventions also calls for its use in combination approaches.

So, while this promising approach thereby has profound cellular effects, it will be important to understand which leukemias are susceptible to this treatment and how this treatment can be combined with complementary strategies to eradicate the disease and improve the prognosis of patients with AML.

## Author contributions

TB, EH and KS conceived the review outline. EH and KS performed the literature search and wrote the draft. EH designed the figures. EH, KS and TB wrote and finalized the manuscript. All authors contributed to the article and approved the submitted version.
